# Ethnicity and attitudes to deceased kidney donation: a survey in Barbados and comparison with Black Caribbean people in the United Kingdom

**DOI:** 10.1186/1471-2458-10-266

**Published:** 2010-05-21

**Authors:** Myfanwy Morgan, O Peter Adams, Paul T Seed, Roger Jones

**Affiliations:** 1 King's College London, Department of Primary Care and Public Health Sciences, Capital House, 42 Weston Street, London SE1 3QD, UK; 2 University of the West Indies, Faculty of Medical Sciences, Cave Hill campus, Barbados

## Abstract

**Background:**

Black minority ethnic groups in the UK have relatively low rates of deceased donation and report a higher prevalence of beliefs that are regarded as barriers to donation. However there is little data from migrants' countries of origin. This paper examines community attitudes to deceased kidney donation in Barbados and compares the findings with a survey conducted in a disadvantaged multi-ethnic area of south London.

**Methods:**

Questionnaires were administered at four public health centres in Barbados and at three private general practices. Adjusted odds ratios were calculated to compare attitudinal responses with a prior survey of 328 Caribbean and 808 White respondents in south London.

**Results:**

Questionnaires were completed by 327 respondents in Barbados (93% response); 42% men and 58% women, with a mean age of 40.4 years (SD 12.6). The main religious groups were Anglican (29%) and Pentecostal (24%). Educational levels ranged from 18% not completing 5th form to 12% with university education. Attitudes to the notion of organ donation were favourable, with 73% willing to donate their kidneys after their death and only 5% definitely against this. Most preferred an opt-in system of donation. Responses to nine attitudinal questions identified 18% as having no concerns and 9% as having 4 or more concerns. The highest level of concern (43%) was for lack of confidence that medical teams would try as hard to save the life of a person who has agreed to donate organs. There was no significant association between age, gender, education or religion and attitudinal barriers, but greater knowledge of donation had some positive effect on attitudes. Comparison of attitudes to donation in south London and Barbados (adjusting for gender, age, level of education, employment status) indicated that a significantly higher proportion of the south London Caribbean respondents identified attitudinal barriers to donation.

**Conclusions:**

Community attitudes in Barbados are favourable to deceased donation based on a system of informed consent. Comparison with south London data supports the hypothesis that the relatively high prevalence of negative attitudes to deceased donation among disadvantaged ethnic minorities in high income countries may reflect feelings of marginalisation and lack of belonging.

## Background

Rates of end stage renal failure are increasing worldwide, leading to greater needs for dialysis and kidney transplantation [1]. Transplantation is cost effective compared with dialysis in the treatment of end stage renal disease and achieves much improved outcomes for patients' quality of life [2]. Currently living kidney donation accounts for about one-third of kidney transplants performed in the UK, with deceased donation forming the main source of kidney transplantation [2].

Black and South Asian populations (from the Indian subcontinent) living in the UK are three to four times as likely to need a kidney transplant compared with the White population. This is associated with higher rates of diabetes and hypertension that are both major causes of end stage renal failure [1,2]. However Black populations in the UK and US have relatively low donation rates [3]. This high need but low supply among minority ethnic populations presents problems in achieving an optimal match of blood group and tissue type where these are less common among the majority population, resulting in patients from minority ethnic groups spending an increased time on the transplantation waiting list [3,4]. Research investigating the lower donation rates among ethnic minorities indicates that although minority ethnic groups generally support the 'gift' of life, they are often less well informed about organ donation and more commonly hold beliefs that are identified as barriers to deceased donation [5-8].

The current research arose through the Centre for Caribbean Health that is run jointly by King's College London and the University of the West Indies (UWI). Through this collaboration we identified a lack of data on attitudes to donation in ethnic minorities' home countries, with this study in Barbados being the first such study in the Caribbean.

Barbados is a middle-income country (GDP per capita $20,200) in the Eastern Caribbean. The 2000 census population (adjusted figure) is 268,792, of which 93.0% are of African origin, 3.2% white and 2.6% mixed (Barbados Statistical Service - personal communication).

Life expectancy at birth in Barbados was 77.0 years in 2007 [9]. However there is a relatively high incidence of diabetes, with an estimated prevalence of 17.5% among people aged 40 years and over, leading to high rates of end stage renal failure [10].

Eight public sector polyclinics strategically located around the island provide free comprehensive primary care. In 2003, 89 private general practitioners provided care based on fee for service, with many people receiving care from both sectors. Provision of dialysis is limited; there is a small unit at the main public hospital and a few dialysis machines in the private sector that cater mainly for tourists. The public dialysis service in Barbados functions at a cost level well below that for high income countries, but is nevertheless relatively expensive in terms of both capital and running costs in relation to health service budgets [11].

Transplantation surgery is occasionally undertaken in Barbados and a few other countries in the English-speaking Caribbean, although Trinidad and Tobago is currently the only country in the Caribbean with a structured system of organ transplantation. Transplantation began in 2006 in Trinidad and Tobago and so far has only involved living donors. However there is interest in establishing a system of deceased donation which would substantially increase the donor pool [12].

This study examines two questions: What are the knowledge and attitudes of persons in Barbados to deceased donation? How do these attitudes and knowledge compare with Black Caribbean counterparts in the UK as recorded in a recent survey in south London? [5].

## Methods

### Design

The Barbados survey was based on a convenience sample of primary care attendees. It involved four of the eight publicly funded health clinics in different parts of the island, covering both urban and rural areas, and three private group general practices in different locations. A quota sampling approach was employed to ensure a mix of gender and age (range 18-65 years). The survey was conducted at both morning and late afternoon/evening sessions.

Comparative data for Black Caribbean and White respondents in London was based on a similar survey we conducted in 2005 among attendees at four large general practices in south London [5].

### Questionnaire

the Barbados questionnaire comprised 2 knowledge questions, 10 attitudinal questions and 9 socio-demographic questions (Additional file 1). The questionnaire was piloted among 14 attendees at health clinics to test its acceptability and comprehensibility in this setting and as a result a few modifications were made. A new question with three options was also added to identify respondents' preference in terms of an organised system of donation.

Two changes were made from the initial south London questionnaire because it was assumed that the Barbados population would be less familiar with organ donation and transplantation. Firstly, the questionnaire was interview administered, as this allowed the fieldworkers to explain about organ donation and transplantation. Secondly, the original 4-point scale for attitudinal statements (strongly agree/agree/disagree/strongly disagree) was simplified to agree/disagree. A further change was that a 'not sure' response category was included for attitudinal questions as we found that 'not sure' or 'don't know' were occasionally written on the south London questionnaires.

The Barbados survey was undertaken during June-August 2008. Recruitment at each health facility involved approaching patients in the waiting area, explaining the purpose of the survey and giving a one-page information sheet to potential respondents. Those agreeing to participate were asked to sign a consent form following full explanation. Community nurses administered the survey in health facilities where they were not employed and also wore their casual clothes, with this approach aiming to ensure that respondents did not give answers that they thought might be expected by health professionals.

### Analysis

Questionnaire data were first entered into Epi-Info and validity checks were undertaken. The data were then transferred to Stata (version 9.2) for analysis. Chi-square and logistic regression analyses were used to test the effects on attitudes to donation of age, education, employment and religion. To compare responses in the Barbados and South London surveys we calculated odds ratios for nine attitudinal questions asked in both surveys, adjusting for gender, and for age, level of education and employment status (each in 3 categories). For logistic regression, the 'event' was always the response that indicated an objection (whether 'agree' or 'disagree'). 'Not sure' in the Barbados questionnaire was regarded as indicating no objection (i.e. no event). When respondents explained the reasons for their response this was usually noted on the questionnaire.

Ethical approval for the Barbados study was granted by the University of the West Indies-Cave Hill/Barbados Ministry of Health Institutional Review Board (February, 2008).

## Results

A total of 327 questionnaires (93% response) were completed in Barbados; 246 (75%) at health clinics and 81(25%) at general practices. Questionnaires each took between 5 and 15 minutes to complete. The main reasons for non-participation were that people were expecting to be called for their appointment, were occupied with children, did not feel well or did not want to be bothered. Twelve questionnaires (3.7%) had some missing data.

### Characteristics of respondents

Respondents comprised 42% men and 58% women, with a mean age of 40.4 years (SD 12.6) (Table 1). Altogether 96% were Black, with others self-identifying as Indian or mixed race. The main religious groups were Anglican (29%) and Pentecostal (24%). The highest educational level completed identified a considerable range; for example, 18% had not completed 5^th ^form whereas 12% had a university education. Altogether 78% of respondents were in either full or part-time employment, 3% were students and 19% were retired, not working or doing occasional work. Analysis of occupations indicated that a small number were in professional posts such as teacher, pharmacist, and accountant. The majority were engaged in routine non-manual occupations, such as clerical officer and receptionist, or in skilled manual occupations such as taxi driver, electrician and chef. A small number were in semi-skilled occupations, such as postman, fisherman and cleaner. Respondents in professional occupations were recruited through general practices and those in semi-skilled occupations through public health clinics. Otherwise there was a considerable mix of occupations among attendees at public health clinics and private general practices.

**Table 1:** Characteristics of respondents in Barbados and south London surveys

	Barbados	South London	
Characteristics		Caribbean	White
	N = 327 %	N = 338 %	N = 808 %
***Gender***			
men	42	32	40
women	58	68	60

***Age -years***			
18-40	49	56	51
40-59	42	32	33
60+	8	12	16

***Religion***			
Anglican	29		
Pentecostal	24		
Seventh Day Adventist	9		
Methodist	7		
Wesleyan/Baptist	5		
All listed above	74	65	58
Other religion	16	5	4
No religion	10	30	38

***Highest education completed***			
Primary education ^a^	18	4	3
High School 5^th/^6^th ^form	47^b^	35	33
Further training	24	44	18
University	12	17	46

***Employment***			
Paid employment	78	49	60
Student	3	21	6
Retired, not working, occasional work	19	30	34

^a ^Many in this group had some secondary education but left school at 16 years before completing 5^th ^form.

^b ^In Barbados 43% respondents had completed 5^th ^form and 4% completed 6^th ^form

Compared with population estimates for Barbados (2008), men were slightly under represented in the sample (42% compared with 48% in population). The age and ethnic distributions were almost identical with the population figures, with the exception of non-representation in the sample of the 3% white population.

### Knowledge and general attitudes to donation

Altogether 30% of respondents had known someone (mainly a relative) with a severe kidney problem. Despite the lack of a transplantation service in Barbados, 20% of respondents were aware that immediately after your death it is possible for your kidneys to be transplanted in someone else, with this knowledge mainly gained from health programmes on North American television channels.

Reported attitudes to the notion of organ donation were very favourable; 73% responded that they would be willing to donate their kidneys after their death and a further 22% were 'not sure', with only 5% definitely against this (Table 2). There were no significant associations between willingness to donate and gender, age, education, religion or occupational status (Table 2). Logistic regression analysis (including age, gender, education and occupation) identified only occupation (working/non-working) as significantly predictive of willingness to donate. The odds ratio (adjusting for age, gender and education) for those not working compared with those working was 0.51, *p = 0*.029 (*CI *0.28-0.93). A further question regarding possible payment to their relative indicated that this was not an important influence on attitudes to donation.

**Table 2:** Socio-demographic characteristics and whether willing to donate organs at death - Barbados survey

	Not willing to donate		**Willing to donate**^**a**^		**Pearson **Χ^**2**^	*P*-value
Characteristics	N	%	N	%		
***Gender***						
Men	4	2.92	133	97.08		
Women	13	6.88	176	93.12		
Total	17	5.21	309	94.79	2.5181	*P = *0.113

***Age-years***						
18-39	8	5.00	152	95.00		
40-59	7	5.04	132	94.96		
60+	2	7.41	25	92.59		
Total	17	5.21	309	94.79	0.2865	*P *= 0.867

***Religion***						
Anglican	3	3.37	86	96.63		
Pentecostal	5	6.76	69	93.24		
Methodist	0	0.00	22	100		
Seventh Day Adventist	0	0.00	27	100		
Catholic	2	18.18	9	81.82		
Nazarine	1	8.33	11	91.67		
Wesleyan/Baptist	1	5.88	11	91.67		
Jehovah's Witness	2	22.22	7	77.78		
Other	0	0.00	21	100		
None	1	3.03	32	96.97		
Total	15	4.76	300	95.24	15.55	*P *= 0.077

***Highest education completed***						
Primary ^b^	4	7.02	53	92.98		
High school 5^th ^form	8	5.84	129	94.16		
High school 6^th ^form	2	16.6	10	83.33		
Other training	1	1.47	67	98.53		
University	1	2.63	37	97.37		
Total	16	4.98	305	95.02	6.8561	*P *= 0.232

***Employment***						
Paid employment	10	3.98	241	96.02		
Student	0	0.00	9	100		
Retired/not working/occasional	6	9.67	56	90.32		
Total	16	4.98	306	95.02	4.0343	*P *= 0.133

^a ^'Not sure' classified with 'willing to' donate. ^b ^See note Table 1

### Specific attitudes to donation

Responses to nine questions concerning specific attitudes to donation were generally positive; 18% had no concerns, 72% had 1 to 3 concerns, and 9% had 4 or more concerns. The highest level of concern and 'not sure' responses were given for the statement '*I am not confident that medical teams would try as hard to save the life of a person who has agreed to donate organs' *(Table 3). This statement also appeared to present some difficulty and required the most thought. Questioning revealed that this was often because respondents were concerned about individual variability in trustworthiness, because 'we are all human' and 'different doctors may act differently'.

**Table 3:** Specific attitudes to donation elicited in Barbados survey (N = 327)

	Percentage responses			Percentage responses
Statement	Agree	Disagree	Not sure	Opposing donation
Is important to me that I could give someone else a chance of life after my death.	95	4	1	4
Donating your organs when you die is a good thing to do.	89	7	5	7
I wouldn't mind who received my kidney after my death.	88	12	1	12
Do not have a problem with my body being cut up after my death.	85	11	3	11
Am not confident that medical teams would try as hard to save the life of a person who has agreed to donate organs.	43	43	14	43
I worry that if I donate my organs for transplant they might be used without my consent for other purposes like medical research.	29	65	6	29
Am concerned that an intact body with no parts removed is needed for the life hereafter.	7	89	4	7
Organ donation is unacceptable because of my religious beliefs.	12	86	2	12
Feel that agreeing when alive to donating kidneys as a gift after death is like tempting death.	9	89	3	9

Neither religious beliefs concerning the body nor a desire to keep the body intact with no parts removed appeared to be major barriers. These beliefs were also not significantly associated with membership of a particular religious group.

Most respondents did not mind who received their kidney. However in considering this question a few respondents described moral criteria, stating that they would not be happy if their kidney was given to a person who had 'done bad things' or who had been in prison.

The attitudes of respondents who were previously aware of deceased donation and transplantation were similar to those who had not previously known about this. The only exception was that 46% of those who already knew about organ donation and transplantation were not confident that medical teams would try as hard to save the life of a person who had agreed to donate their organs, compared with 69% who had not known about donation (difference 23%, 95% CI -37% to -8%, *p *= 0.0034). Those who had previous knowledge of deceased donation were also rather less likely to be concerned about their body being cut after their death compared with those who had not previously known (90% v 82%, difference 18%, CI -3% to 18%).

There was no significant association between age, gender, education or religion and individual attitudinal barriers. However, 76% of respondents with fewer than 4 concerns were willing to donate compared with 42% with 4 or more concerns.

### Comparison with South London

The south London sample included 338 Black Caribbean and 808 White primary care attendees [5] (Table 1).

In terms of knowledge, a significantly higher proportion of south London respondents were aware of the possibility of leaving their kidneys for transplant in someone else after their death; 94% White and 87% Black Caribbean respondents in the south London survey compared with the 20% of the Barbadian respondents.

Comparison of attitudes to donation in South London and Barbados (adjusting for gender, age, level of education and employment status) indicated that a significantly higher proportion of south London Caribbean respondents gave responses to attitudinal questions that identified barriers to donation (Table 4). The only attitudinal question responded to more positively by the south London Caribbean respondents was feeling confident that medical teams would try as hard to save the life of a person who has agreed to donate organs.

**Table 4:** Percentage giving responses opposed to donation to statements in the Barbados survey and South London, odds ratios (adjusting for gender, age, education and employment status), 95% CI and *p*-value

	Barbados N = 327	South London Caribbean N = 338	South London White N = 805	South London Caribbean N = 338	South London White N = 805
**Statement**	**% responses opposed to ** **kidney donation**	**% responses opposed to ** **kidney donation**	**% responses opposed to ** **kidney donation**	**Odds ratio** **95% CI^a^** ***p *value**	**Odds ratio** **95% CI^a^** ***p *value**

Is important to me that I could give someone else a chance of life after my death. (% Disagree)	4.0	15.2	4.4	4.79 2.14 to 9.51 *p *< 0.001	1.31 0.64 to 2.68 *p *= 0.45
Donating your organs when you die is a good thing to do. (% Disagree)	6.5	24.0	3.9	5.18 2.93 to 9.1 *p *< 0.001	0.765 0.40 to 1.42 *p *= 0.38
I wouldn't mind who received my kidney after my death. (% Disagree)	11.8	24.7	9.1	.39 .24- to .64 *p *< 0.001	1.29 0.81 to 2.08 *p *= 0.25
Am not confident that medical teams would try as hard to save the life of a person who has agreed to donate organs ^b ^(% Agree)	43.4	20.3	6.2	.34 .23 to .51 *p *< 0.001	.10 0.06 to .15 *P *< 0.001
I worry that if I donate my organs for transplant they might be used without my consent for other purposes like medical research. (% Agree)	29.0	62.7	43.4	4.50 3.08 to 6.57 *p *< 0.001	2.56 1.86 to 3.51 *P *< 0.001
Concerned that an intact body with no parts removed is needed for the life hereafter ^c ^(% Agree)	7.1	39.3	16.0	7.90 4.62 to 13.5 *p *< 0.001	3.74 2.20- to 6.29 *P *< 0.001
Organ donation is unacceptable because of my religious beliefs ^d ^(% Agree)	12.1	18.9	5.0	1.98 1.21 to 3.26 *p *< 0.001	0.55 0.33 to 0.94 *p *= 0.02
Feel that agreeing when alive to donating kidneys as a gift after death is like tempting death ^e ^(% Agree)	8.5	35.2	19.3	6.35 3.77 to 10.69 *p *< 0.001	2.78 1.74 to 4.45 *p *< 0.001

a Barbados as reference group.

b In South London the equivalent question was "Am confident" ......

c In South London the equivalent question did not include 'for the life hereafter'

d In South London the equivalent question was "Donating my organs is acceptable to my religious beliefs."

e In South London the equivalent question referred to carrying a donor card.

## Discussion

This study is the first to examine attitudes to deceased donation in the Caribbean and indicated that attitudes to donation in Barbados were generally positive, with few religious or specific cultural barriers. There were also no differences in attitudes to donation by age, gender or education, although increased knowledge of the possibilities of donation and transplantation had some positive effect on attitudes. The only question for which a large proportion of Barbadian respondents identified a potential barrier to donation was in relation to trust in medical teams and health care system. Questioning indicated that respondents' worries about this mainly reflected their concerns about individual variability, including possible variability among health professionals. This contrasts with a more fundamental distrust of medicine and the health system that appears to be increasingly prevalent in high-income countries [13].

The significantly higher prevalence of worries and concerns regarding deceased donation among Caribbean respondents in south London compared with Barbados held after adjusting for socio-demographic characteristics. There were some differences in response categories between the two surveys. However as responses were analysed as dichotomous agree/disagree this would reduce any effects of the larger number of scaled response categories in south London. Responses recorded as 'not sure' were more common in Barbados, probably influenced by the inclusion of this category rather than merely noting uncertainty on the questionnaire as in south London. 'Not sure' in each case was regarded as indicating no objection, thus possibly increasing favourable responses. However, grouping 'not sure' with 'not willing' to donate did not produce significant differences in the responses presented in Table 2. For specific attitudes to donation the percentage of 'not sure responses' was less than 7%, with the only exception of 14% giving a 'not sure' response to a statement regarding confidence that medical teams would try as hard to save the life of a person who has agreed to donate organs (Table 3).

Respondents of Caribbean origin in south London were predominately from Jamaica and may therefore have differed in their cultural attitudes compared with migrants from Barbados. It would be interesting to investigate this and to undertake a similar survey of attitudes to organ donation among primary care attendees in Jamaica. However we hypothesise that the more negative attitudes identified among ethnic minorities in south London may arise not so much from cultural differences as from feelings of marginalisation that are associated with membership of a minority ethnic group, particularly when combined with low socio-economic status. This hypothesis is supported by a qualitative study of the factors associated with low rates of participation by the African Caribbean population in local community networks in a multi-ethnic area of south London [14]. A key factor accounting for the low participation among African Caribbean respondents was identified as a lack of trust and confidence in formal organisations, with their social relationships and participation therefore mainly occurring within their own community networks [14]. Our previous in-depth study with people of Caribbean origin (predominately from Jamaica) living in relatively disadvantaged multi-ethnic areas of South London also indicated that these respondents did not regard themselves as fully accepted and integrated into British society. For example, they described experiences of discrimination and disadvantage in relation to the educational system, job opportunities and the police, and referred to the existence of several 'societies' rather than seeing themselves as part of a single unified society [15]. These feelings of marginalisation and lack of belonging to British society were associated with an idealised desire to return home to the Caribbean for burial with their body 'whole', or at least to be buried in this way following a Caribbean style funeral in the UK. Funerals and burial thus appeared to have a symbolic value in reconciling the experience of a divided identity in life [15].

In contrast to the situation of disadvantaged minority ethnic groups in the UK, British and Irish emigrants to south-eastern Spain have been shown to express more positive attitudes to organ donation compared with the local community [16]. This migrant group is however described as relatively affluent and 'perfectly integrated into the social structure', rather than experiencing the feelings of marginalisation that characterise disadvantaged minority ethnic groups.

Further evidence to support the notion that social position and feelings of marginalisation shape attitudes and participation in civic society is provided at a societal level by evidence of a relationship among high income countries between increased income inequality and a decline in public trust [16]. Wilkinson and Pickett hypothesise that it is income inequality that affects trust, and that in a society where there is a high level of trust this leads to people feeling secure, worrying less and viewing others as co-operative rather than competitive [17]. Socio-economically disadvantaged members of minority ethnic groups living in high income countries with considerable income inequality may thus experience a reduced sense of trust in the health system and less desire to act altruistically for the public good, whereas citizens of more equal countries have a greater public trust that is likely to be reflected in a greater willingness to give for the public good.

This research, like the south London study, was based on attendees at primary care facilities. It is possible that attendance at primary care sensitises people to health issues and may also over-represent people with chronic health conditions. Despite these limitations, primary care is often regarded as a suitable and low cost sampling frame. This reflects the high proportion of the population who attend over a year, with attendance occurring for acute and preventive/administrative reasons as well as for chronic health problems. The selection of respondents in both Barbados and the UK from primary care centres also means that any bias would also be present in both samples, thus not invalidating the comparison.

## Conclusions

The study indicates that Barbados has a positive social environment for setting up or participating in a wider Caribbean transplantation programme based on the 'gift' of life, with an overwhelming preference for an opt-in system that is practised in the UK.

The data also support the hypothesis that relatively negative attitudes to organ donation reported by disadvantaged minority ethnic groups in the UK, US and other high income countries are associated with feelings of lack integration and belonging. Champions from minority groups may thus be important in increasing trust and have a positive influence on attitudes to donation among migrant communities.

## Competing interests

The authors declare that they have no competing interests.

## Authors' contributions

MM, RJ and OPA designed the Barbados study, MM and OPA oversaw the fieldwork, PS undertook the statistical analysis, MM interpreted the data and wrote the paper with all authors contributing. All authors read and approved the final manuscript.

## Author information

Myfanwy Morgan is Professor of Medical Sociology, Paul Seed is Senior Lecturer in Medical Statistics, Roger Jones is Wolfson Professor and Head of General Practice, all at King's College London. O. Peter Adams is Lecturer in Family Medicine and Deputy Dean Phase 1, University of the West Indies, Barbados.

## Pre-publication history

The pre-publication history for this paper can be accessed here:

http://www.biomedcentral.com/1471-2458/10/266/prepub

## Supplementary Material

Additional file 1 **Attitudes to organ donation - Barbados questionnaire**. Questionnaire completed by respondents in Barbados Click here for file
