# Supplementary material for: Ethnicity and attitudes to deceased kidney donation: a survey in Barbados and comparison with Black Caribbean people in the United Kingdom
Source: BMC Public Health. 2010 May 21;10:266. doi: 10.1186/1471-2458-10-266 (PMC2893092; doi:10.1186/1471-2458-10-266)
Supplement: Additional file 1 — Attitudes to organ donation - Barbados questionnaire. Questionnaire completed by respondents in Barbados [file 1471-2458-10-266-S1.DOC]

#
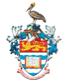

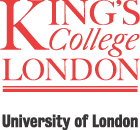
 University of the West Indies

# *Kidney Donation Project Group*

Professor Myfanwy Morgan Dr Peter Adams

Professor Roger Jones

# Barbados Questionnaire

# Attitudes to kidney donation and transplantation

# This Questionnaire asks your views on kidney donation.

Everything you tell us will be treated as **strictly confidential.**

#### Thank you very much for your help.

# Study no.

# (C) For permission to use questionnaire please contact myfanwy.morgan@kcl.ac.uk

# Section A

###

**1.** Do you know anyone who has severe kidney problems?

Yes  relative  friend

No

2. Did you know that immediately after someone dies it is possible for their kidneys to be removed and transplanted in somebody else whose own kidneys are not working?

### Yes

*No*

**3.** What do you think about donating your kidneys to be transplanted in someone else after you die? Would you be willing to donate your kidneys after you die?

Yes

Not sure

No

**4.** In some countries it is necessary to gain approval from a deceased persons family to remove their kidneys. In other countries it is lawful for doctors to remove kidneys from any adult who has just died, unless that person had forbidden it while they were alive.

What sort of system do you think should be should be introduced in Barbados?

Doctors able to remove kidneys unless patient registers that do not wish this

Doctors only able to remove kidneys if patients’ family agrees

I am not sure how I feel about it

#### Section B

# I will now read some statements and would like to know whether you agree or disagree with it. There are no right or wrong answers, we just want to know peoples views.

**5.** If I donated my kidneys I would not mind who received my kidney after my death.

Agree

Disagree

**6.** It would be important to me to know that I could give someone else a chance of life after my

death by donating my kidneys.

Agree

Disagree

**7.** I trust doctors and would not worry if they were allowed to remove my kidneys when I die.

Agree

Disagree

**8.** I do not have a problem with my body being cut after my death and the kidneys removed.

Agree

Disagree

**9.** I regard donating my kidneys as being a way of serving God.

Agree

Disagree

**10.** I worry that if I agree to donate my kidneys for transplant, they might be used without my

consent for medical research.

Agree

Disagree

**11.** Some people feel that agreeing when they are alive to donating their kidneys as a gift when they die is like tempting death. What do you think?

Agree

Disagree

**12.** Some people are concerned that an intact body with no parts removed is needed for the life hereafter. What do you think?

Agree

Disagree

**13.** Overall do you agree that donating your organs when you die is a good thing to do.

Agree

Disagree

#### Section C

#### Finally, some information about you to help us analyse the survey data

**14.** Were you born in Barbados?

Yes  No  please describe where: ________________

**15.** What is your ethnic group?:

­

Black  Asian

White  Other  specify……………………………………….

Mixed

**16.** What is the highest level of education you have completed? Please **tick** one

Primary education

### High school 5th form

High school 6th form

Community College

Polytechnic

University education

Other (please describe……………………………………………………

**16.** Do you have any type of paid work (either full-time, part-time or occasional) or are you not working? – Please **tick** one

Yes - Full-time  No - Retired

Part-time  Student

Occasional  Look after house / children

Looking for work

**If yes**, what do you do? Please give job title and describe what you do: __________________________________________________________________________

__________________________________________________________________________

**17.** Do you (or your partner) own a car or van? Yes  No

**18.** Do you have a television in your home? Yes  No

**19.** What is your religion? Anglican …………..

Pentecostal ………

Methodist ……….

Seventh Day Adventist

Roman Catholic …...

None ………………

Other ……………….

(please describe)…………………………………………..

/ /

**20.** What is your date of birth?

D M Y

**21.** Are you?

Male

### Female

##### Thank you for your assistance
